# Mapping morbidity 10 years prior to a diagnosis of young onset Alzheimer's disease

**DOI:** 10.1002/alz.13681

**Published:** 2024-01-31

**Authors:** Line Damsgaard, Janet Janbek, Thomas M. Laursen, Peter Høgh, Karsten Vestergaard, Hanne Gottrup, Christina Jensen‐Dahm, Gunhild Waldemar

**Affiliations:** ^1^ Danish Dementia Research Centre Department of Neurology Copenhagen University Hospital – Rigshospitalet Copenhagen Denmark; ^2^ National Centre for Register‐based Research Department of Economics and Business Economics Aarhus University Aarhus Denmark; ^3^ Department of Neurology Zealand University Hospital Roskilde Denmark; ^4^ Department of Clinical Medicine University of Copenhagen Copenhagen Denmark; ^5^ Dementia Clinic Department of Neurology Aalborg University Hospital Aalborg Denmark; ^6^ Dementia Clinic Department of Neurology Aarhus University Hospital Aarhus Denmark

**Keywords:** Alzheimer's disease, early warning signs, epidemiology, morbidity, registry‐based, young onset dementia

## Abstract

**INTRODUCTION:**

Early symptoms in young onset Alzheimer's disease (YOAD) may be misinterpreted, causing delayed diagnosis. This population‐based study aimed to map morbidity prior to YOAD diagnosis.

**METHODS:**

In a register‐based incidence density matched nested case‐control study, we examined hospital‐diagnosed morbidity for people diagnosed with YOAD in Danish memory clinics during 2016‐2020 compared to controls in a 10‐year period. Conditional logistic regression produced incidence rate ratios (IRRs).

**RESULTS:**

The study included 1745 cases and 5235 controls. YOAD patients had a higher morbidity burden in the year immediately before dementia diagnosis, for certain disorders up to 10 years before. This was especially evident for psychiatric morbidity with the highest increased IRRs throughout the entire period and IRR 1.43 (95% confidence interval 1.14–1.79) in the 5–10‐years before dementia diagnosis.

**DISCUSSION:**

YOAD patients display a different pattern of morbidity up to 10 years prior to diagnosis. Awareness of specific alterations in morbidity may improve efforts toward a timely diagnosis.

**Highlights:**

- Retrospective, nested case‐control study of young onset Alzheimer's disease (YOAD).
- YOAD cases had a higher morbidity burden than controls.
- YOAD cases had a higher psychiatric morbidity burden up to 10 years before diagnosis.
- Altered morbidity patterns could serve as an early warning sign of YOAD.

## 1 BACKGROUND

While several studies have sought to describe the earliest symptoms of Alzheimer's disease (AD),^1^ to our knowledge, no previous studies have broadly explored morbidity prior to a diagnosis of young onset AD (YOAD). Studies examining morbidity prior to an AD or all‐cause dementia diagnosis usually target one prespecified disease or symptom, often as risk studies, as outlined in the 2020 Lancet Commission's report on dementia prevention, intervention, and care.^2^ Here, evidence was gathered on, among others, hearing impairment, traumatic brain injury, hypertension, diabetes, cardiovascular disease, depression, and sleep disorders and subsequent dementia. Other studies have found associations between many diverse diseases and symptoms and a future diagnosis of all‐cause dementia, such as infectious diseases,^3^ inflammatory bowel disease,^4^ stress diagnoses,^5^ anxiety,^6^ and so forth. Further studies that stratify on etiology are needed, as (co)morbidity burden is likely heterogeneous between different causes of dementia. Furthermore, the vast majority of studies on risk factors and early symptoms in AD have focused on late onset AD (LOAD). As morbidity generally tends to increase with increasing age, there is likely a difference in the pattern of morbidity for those diagnosed with YOAD compared to those diagnosed with LOAD even based on age alone. Therefore, the knowledge on morbidity preceding LOAD is unlikely to be directly transferable to YOAD patients.

Due to the risk of reverse causation, it is often challenging to establish whether a disease or symptom is a risk factor for dementia or an early manifestation of the dementia disorder. Regardless, increased knowledge on such diseases and symptoms may serve as early warning signs. In a study of pre‐diagnostic symptoms of 89 young‐onset dementia patients, only 5% of patients reported cognitive symptoms five years before diagnosis.^7^ Establishing key features of the pre‐diagnostic phase of YOAD, including both cognitive and non‐cognitive symptoms, may help patients, caregivers, and especially general practitioners (GPs) recognize the need for memory clinic referral during the early phase of clinical deterioration. Studies have shown a large diagnostic delay in young onset dementia, which may have detrimental effects on work life, financial stability, and family roles.^8^, ^9^ Thus, minimizing diagnostic delay is key in ensuring timely diagnosis and support. Furthermore, an early diagnosis allows for timely initiation of treatment, which may be especially important with advances in early biomarkers and the emergence of disease‐modifying treatments.

In a previous study, we demonstrated that YOAD patients have increased healthcare utilization from 10 years before diagnosis,^10^ and the present study elaborates on those findings.

**RESEARCH IN CONTEXT**

1. **Systematic review**: The authors searched PubMed for literature on dementia and prior morbidity. Only few studies were found, focusing on either late onset Alzheimer's disease or young onset all‐cause dementia.
2. **Interpretation**: This study demonstrated a higher morbidity burden among patients with young onset Alzheimer's disease (YOAD) than controls, detectable up to as long as 10 years before dementia diagnosis. There was a substantial increase in psychiatric diagnoses, supporting prior reports on the significance of stress and depression in the years leading up to diagnosis.
3. **Future directions**: Altered hospital‐diagnosed morbidity patterns in young patients with cognitive complaints may be an early warning sign of YOAD. Future studies should further explore early symptoms and morbidity prior to a diagnosis of YOAD by exploring consultations in the general practice setting and the use of prescription medication. This approach may help to identify patients at high risk of YOAD earlier than today.

The aim of this study was to explore whether individuals diagnosed with YOAD differ from cognitively healthy matched adults in terms of reasons for hospital contacts 10 years preceding diagnosis, in order to describe potential patterns that characterize the prodromal phase of YOAD.

## 2 METHODS

### 2.1 Data sources

Cases were identified from the Danish Quality Database for Dementia (DanDem). The database was established in 2016, and it is mandatory for all secondary healthcare facilities that accept referrals for diagnostic evaluation of cognitive impairment and dementia to enter information in DanDem upon completion of diagnostic evaluation. Thus, DanDem contains information on all patients seen at Danish memory clinics from 2016 onward. Variables included are, among others, etiology, severity of the dementia syndrome at time of diagnosis (if applicable), and diagnostic investigations performed, including results of selected cognitive tests. Furthermore, we used the Danish National Patient Registry (DNPR) and the Danish Psychiatric Central Research Register (DPCRR), which contain information on all somatic and psychiatric diagnoses given in the secondary healthcare sector since 1977 and 1969, respectively, with outpatient data added in 1995.^11^, ^12^ The Danish National Prescription Registry (DNPrR)^13^ was used to draw information on filled prescription medication used in the censoring of potential controls. Covariates were identified from the Population Education Register^14^ and the Civil Registration System, with the latter also used for accurate linkage between the registers through each Danish citizen's unique 10‐digit personal identification number.^15^

### 2.2 Study design and population

We conducted a retrospective incidence density matched nested case‐control study following the approach of our previous study on YOAD.^10^ Cases included all individuals diagnosed with mild cognitive impairment (MCI) or dementia due to AD (YOAD) in a Danish memory clinic between 2016 and 2020. Matched controls were drawn from a nationwide cohort of all Danish citizens.

#### 2.2.1 Case definition

By convention, YOAD is defined as dementia due to AD with symptom onset before age 65 years. As information on the time of symptom onset for patients was not available, we approximated symptom onset by including all patients diagnosed with mild cognitive impairment (MCI) or dementia due to AD before age 70 years, assuming a 5‐year time lag between symptom onset and final diagnosis, as prior research has shown mean time to diagnosis around 5 years for YOAD patients.^8^, ^9^ The AD diagnosis was made according to the National Institute on Aging‐Alzheimer's Association workgroups (NIA‐AA) criteria,^16^ and disease severity was determined based on International Classification of Diseases (ICD) 10 criteria.^17^ Thus, cases were individuals with a first diagnosis of MCI or dementia with AD etiology in DanDem from the start of the register (2016) through 2020. Index date was the date of diagnosis (and the date of matching for controls).

#### 2.2.2 Control definition

Each case was matched with three controls drawn from the full risk set, the entire Danish population, on age and sex. Each control was at risk for YOAD at matching date. Thus, incidence density matching was used, allowing odds ratios from conditional logistic regression analyses to be interpreted as incidence rate ratios (IRR).^18^ Controls were randomly selected with the following criteria: (1) no entry in DanDem before index date (never referred to or seen at a memory clinic), (2) no MCI or dementia diagnosis in DNPR or DPCRR before index date, (3) no redeemed prescription for antidementia medication in DNPrR before index date. ICD codes for identification of MCI and dementia diagnosis in DNPR/DPCRR and anatomical therapeutic chemical (ATC) codes for identification of dementia medication in DNPrR can be found in Table S1.

#### 2.2.3 Overall censoring criteria

Individuals with Down syndrome (ICD‐8: 759.3, ICD‐10: DQ90) and Mental Retardation (ICD‐8: 311‐315, ICD‐10: DF70‐DF79) were censored. To ensure completeness of data, cases and controls who did not live in Denmark throughout the study period were also censored. As incidence density matching was used, controls were censored if they had a dementia diagnosis or a redeemed prescription of antidementia medication prior to diagnosis. Censoring criteria and specific diagnostic codes used to determine these can be seen in Table S1.

### 2.3 Definition of morbidity

Primary and secondary discharge diagnoses from in‐ and outpatient hospital contacts were drawn from DNPR and DPCRR. Diagnoses were grouped according to overall categories of diseases and health problems, based on chapters in the ICD‐10 classification (chapters I‐XIV, XVIII‐XIX, and XXI). The study population was considered exposed if they had at least one diagnosis within each of these categories during the 10 years prior to the index date. All contacts related to pregnancy, childbirth or the perinatal period, and congenital malformations and chromosomal abnormalities (ICD‐10 chapters XV, XVI, XVII) and procedural codes (ICD‐10 chapter XX) were excluded. As any prior MCI or dementia diagnosis was a censoring criterion for controls, these ICD‐10 codes (Table S1) were censored.

### 2.4 Covariates

Analyses were adjusted for age, sex, highest attained educational level at age 40 years (or at time of diagnosis, whichever came first), and civil status at index date.

### 2.5 Data analysis

#### 2.5.1 Main analysis

For each disease category we used a conditional logistic regression model to investigate the association between having at least one diagnosis within the corresponding ICD‐10 chapter and subsequent diagnosis of YOAD. Each matched set served as a stratum in the regression model. IRRs were calculated (a) for the entire 10‐year period, (b) for diagnosis in the 10 ‐ >5‐year interval prior to index date, (c) for the 5 ‐ >1‐year interval prior to index date, and (d) for the ≤1‐year interval prior to index date, to investigate latency between symptoms/diagnoses and dementia diagnosis. In addition, the three most prevalent diagnoses (on a two‐digit level, e.g., A01) within each disease category in the study population were identified, and the association between each specific diagnosis and YOAD was investigated.

#### 2.5.2 Sensitivity analysis

In a sensitivity analysis, patients were grouped by YOAD disease severity, examining those with MCI/mild dementia or moderate/severe dementia, compared with their respective matched controls. In another sensitivity analysis, all contacts in the 6 months preceding the index date were censored. Furthermore, sensitivity analyses were performed stratifying cases by sex and by age at diagnosis (age < 55 years, age ≥ 55 years).

#### 2.5.3 Post hoc analysis

Based on the main results, post‐hoc analyses were done investigating the association between ICD‐10 codes F00‐F09 (Organic, including symptomatic, mental disorders) (excluding MCI or dementia diagnoses, see Table S1), F10‐19 (Mental and behavioral disorders due to psychoactive substance use), F20‐29 (Schizophrenia, schizotypal and delusional disorders), F30‐39 (Mood [affective] disorders), F40‐F48 (Neurotic, stress‐related and somatoform disorders), F50‐59 (Behavioral syndromes associated with physiological disturbances and physical factors), F60‐69 (Disorders of adult personality and behavior), and F99 (Unspecified mental disorder) and subsequent diagnosis of YOAD.

Analyses were presented in two adjustment models; one unadjusted, and one adjusted for age, sex, highest attained educational level at age 40 years (or at time of diagnosis, whichever came first), and civil status at index date. A 5% significance level was applied for all analyses.

Data management was performed using SAS 9.4 software. This research project was approved by the Danish Data Protection Agency, Statistics Denmark, and the Danish Health Data Authority. Danish law does not require ethics committee approval or informed patient consent.

## 3 RESULTS

**FIGURE 1:**
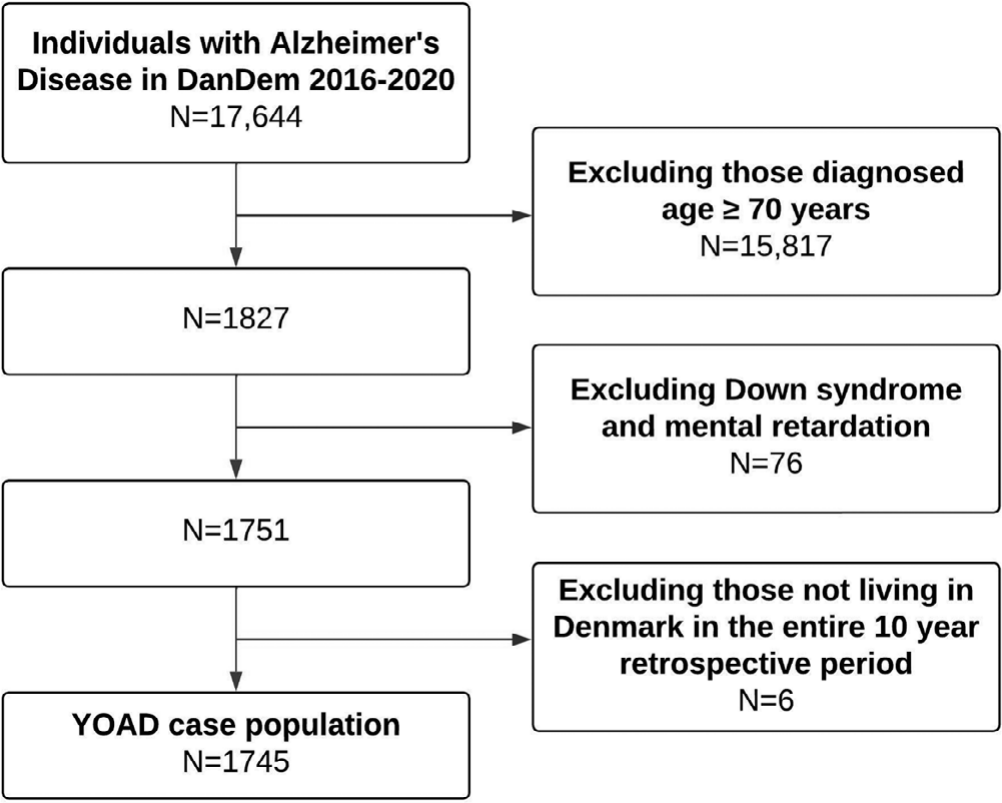
Flowchart of case selection. DanDem: Danish Quality Database for Dementia, YOAD, young onset Alzheimer's disease.

There were 17,644 individuals with a diagnosis of AD in DanDem between 2016 and 2020. Of these, 1827 were diagnosed before age 70 years. After censoring, there were 1745 cases (Figure 1). The mean age at diagnosis was 64.5 years, and 58% of the patients were female (Table 1). There were 63% of the cases diagnosed with either MCI or mild dementia at time of diagnosis, and 37% diagnosed with moderate/severe dementia. The covariates were evenly distributed among cases and controls.

**TABLE 1:** Baseline characteristics of study population.

	Cases *n* = 1745	Controls *n* = 5235
Age at index date, mean years (SD) [range]	64.5 (5.1) [35.8–69.9]	64.5 (5.1) [35.5–69.9]
Sex, male/female, *n* (%)	726 (42%)/1019 (58%)	2178 (42%)/3057 (58%)
Dementia syndrome severity at time of diagnosis, *n* (%)		
Mild cognitive impairment	84 (5%)	–
Mild dementia	1010 (58%)	–
Moderate dementia	530 (30%)	–
Severe dementia	121 (7%)	–
Cognitive examination scores at first visit,^a^ mean (SD)		
MMSE	21.0 (5.5)	–
ACE	65.2 (15.4)	–
Educational attainment at age 40, *n* (%)		
Low	1184 (68%)	3629 (69%)
Medium	374 (21%)	1118 (21%)
High	104 (6%)	268 (5%)
Unknown	83 (5%)	220 (5%)
Civil status at index date, *n* (%)		
Married	1094 (63%)	3377 (65%)
Divorced	321 (18%)	869 (17%)
Widowed	133 (8%)	349 (7%)
Never married	177 (10%)	611 (12%)
Unknown	20 (1%)	29 (1%)
Individuals with ≥1 diagnosis within each category, *n* (%)		
Certain infections	123 (7%)	365 (7%)
Neoplasms	363 (21%)	1065 (20%)
Hematological/immunological	67 (4%)	161 (3%)
Endocrine/metabolic	446 (26%)	1042 (20%)
Mental and behavioral^b^	389 (22%)	428 (8%)
Nervous system^b^	307 (18%)	647 (12%)
Eye and adnexa	258 (15%)	660 (13%)
Ear and mastoid process	188 (11%)	409 (8%)
Circulatory system	589 (34%)	1516 (29%)
Respiratory system	245 (14%)	658 (13%)
Digestive system	456 (26%)	1355 (26%)
Skin/subcutaneous system	168 (10%)	436 (8%)
Musculoskeletal system	689 (39%)	2465 (41%)
Genitourinary system	375 (21%)	1037 (20%)
Symptoms/signs not classified elsewhere	999 (57%)	1842 (35%)
Injuries, poisonings, external causes	896 (51%)	2331 (45%)
Factors influencing health status	1721 (99%)	4723 (90%)

*Note*: Where percentages do not add up to 100%, this is due to rounding up/down.

Abbreviation: Sd, standard deviation.

^a^ MMSE, Mini‐Mental State Examination (reliable information for 1590 YOAD patients); ACE, Addenbrooke's Cognitive Examination (reliable information for 1173 YOAD patients).

^b^ Excluding dementia diagnosis.

**FIGURE 2:**
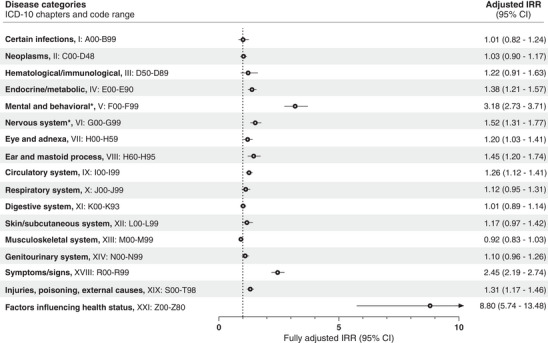
Incidence rate ratios (IRRs) for young onset Alzheimer's disease are plotted by disease categories in the 10‐year retrospective study period. For the refence group (dementia‐free controls), the IRR is equal to 1 (as indicated by the dotted vertical line). Error bars represent 95% confidence intervals (CI). The IRRs presented are adjusted for age, sex, highest attained educational level at age 40 years (or at time of diagnosis, whichever came first), and civil status at index date. Unadjusted estimates are presented in Table S2. * Excluding mild cognitive impairment and dementia diagnoses. ICD: International Classification of Diseases.

Figure 2 shows the overall adjusted IRRs for a diagnosis within the disease categories in YOAD cases compared to controls (unadjusted IRRs in Table S2). The highest overall increase was found for the categories Factors influencing health status with an IRR of 8.80 (95% confidence interval [CI] 5.74–13.48), Mental and behavioral disorders (IRR 3.18, 95% CI 2.73–3.71), and Symptoms/signs not classified elsewhere (IRR 2.45, 95% CI 2.19–2.74).

**FIGURE 3:**
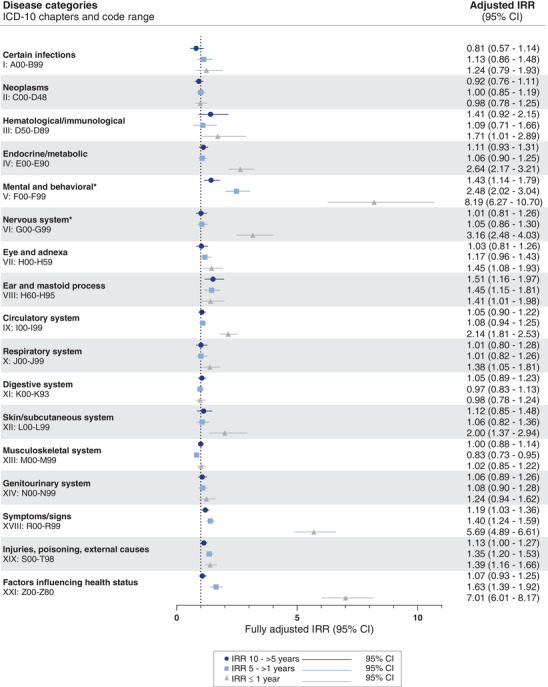
Incidence rate ratios (IRRs) for young onset Alzheimer's disease are plotted by disease categories in three time‐intervals prior to diagnosis. For the refence group (dementia‐free controls), the IRR is equal to 1 (as indicated by the dotted vertical line). Error bars represent 95% confidence intervals (CI). The IRRs presented are adjusted for age, sex, highest attained educational level at age 40 years (or at time of diagnosis, whichever came first), and civil status at index date. Unadjusted estimates are presented in Table S2. * Excluding mild cognitive impairment and dementia diagnoses. ICD: International Classification of Diseases.

When looking at the retrospective period 10 ‐ >5 years prior to index date (Figure 3), significantly increased IRRs were only found for a few disease categories, with the most notable increases found in Diseases of the ear and mastoid process (IRR 1.51, 95% CI 1.16–1.97) and Mental and behavioral disorders (IRR 1.43, 95% CI 1.14–1.79). In the 5 ‐ >1 year prior to index date, significantly increased IRRs were found for Mental and behavioral disorders, Diseases of the ear and mastoid process, Symptoms/signs not classified elsewhere, Injuries, poisonings, and other external causes, and Factors influencing health status, with Mental and behavioral disorders the disease category with the highest IRR (2.48, 95% CI 2.02–3.04).

In the period immediately preceding index date, 12 out of the 17 disease categories investigated showed significantly increased IRRs. In a sensitivity analysis (Table S3), all contacts registered during the 6 months before index date were censored, thus investigating the time period ≤1 year–6 months prior to index date; IRRs for all but Hematological/immunological diseases, Diseases of the eye and adnexa, Diseases of the ear and mastoid process, and Diseases of the respiratory system remained significantly increased.

**FIGURE 4:**
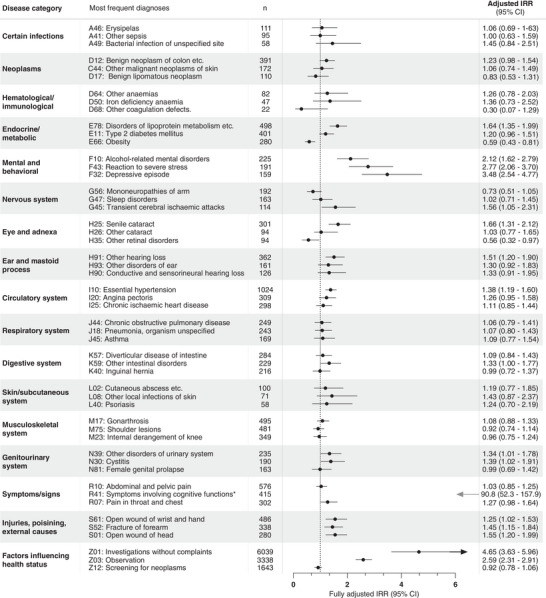
Incidence rate ratios (IRRs) for young onset Alzheimer's disease are plotted for the three most frequent diagnoses in the study population in each disease category. For the refence group (dementia‐free controls), the IRR is equal to 1 (as indicated by the dotted vertical line). Error bars represent 95% confidence intervals (CI). The IRRs presented are adjusted for age, sex, highest attained educational level at age 40 years (or at time of diagnosis, whichever came first), and civil status at index date. Unadjusted estimates are presented in Table S4. * Note that for the diagnostic code R41, estimate and CI is outside the graph limits.

Figure 4 shows the three most frequent diagnoses within each disease category in the entire study population and corresponding IRRs (unadjusted IRRs in Table S4). The largest increase was found for the ICD‐10 code R41 (symptoms involving cognitive functions) in the category Symptoms/signs not classified elsewhere with an IRR of 90.84 (95% CI 52.29–157.87) (of cases, 23% had been given this diagnosis at least once during the study period, and for controls this was 0.3%) and for the code Z01 (other special examinations and investigations of persons without complaint or reported diagnosis) in the category Factors influencing health status with an IRR of 4.65 (95% CI 3.63–5.96). The three most prominently increased IRRs for specific diseases/disorders were found for the three psychiatric codes: F10 (mental and behavioral disorders due to use of alcohol), F43 (reaction to severe stress and adjustment disorders), and F32 (depressive episode).

**FIGURE 5:**
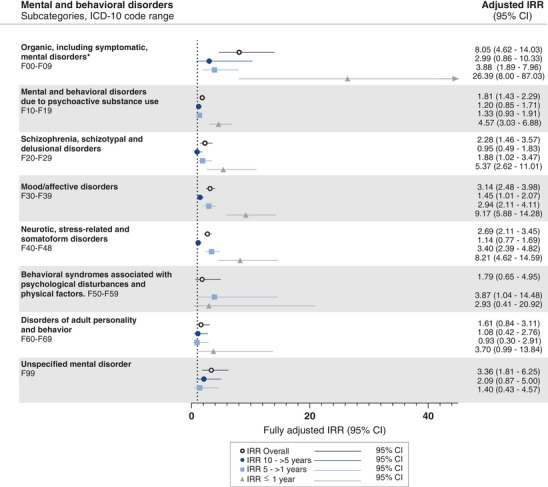
Incidence rate ratios (IRRs) for young onset Alzheimer's disease are plotted by subcategories of mental and behavioral disorders prior to diagnosis. For the refence group (dementia‐free controls), the IRR is equal to 1 (as indicated by the dotted vertical line). Error bars represent 95% confidence intervals (CI). The IRRs presented are adjusted for age, sex, highest attained educational level at age 40 years (or at time of diagnosis, whichever came first), and civil status at index date. Where no estimates are presented, there were too few events to analyze. Unadjusted estimates are presented in Table S6. ICD: International Classification of Diseases. * Excluding mild cognitive impairment and dementia diagnoses.

In the post‐hoc analysis exploring the association between relevant Mental and behavioral disorders’ subcategories and YOAD, IRRs were significantly increased across nearly all subcategories, most prominently F00 (Organic, including symptomatic, mental disorders) (Figure 5). In all performed analyses, adjustment generally did not impact the estimates (Tables S2‐S6).

We conducted a sensitivity analysis dividing the study population by dementia syndrome severity, grouping cases with MCI/mild dementia and moderate/severe dementia, and comparing them to their respective control groups. While the magnitude of the associations differed somewhat, the overall patterns observed were generally similar to those found in the main analysis (Table S5). A sensitivity analysis stratifying YOAD patients by age and sex yielded similar results as in the main analysis, as did removing those with MCI (results not shown).

## 4 DISCUSSION

In this nested case‐control study, we found that YOAD patients had a higher morbidity burden than age‐ and sex‐matched controls, particularly in the year immediately preceding AD diagnosis, and for certain disorders up to 10 years prior. This was especially evident for psychiatric morbidity, following which the likelihood of AD diagnosis was increased by 43% if diagnosed in the 5‐ to 10‐year interval prior to YOAD. Among the most frequent psychiatric diagnoses in the study population were stress and depression, both of which increased the likelihood of YOAD diagnosis. In an analysis of psychiatric subcategories, all of these had significantly increased IRRs, though as this was a post‐hoc analysis, these results should be interpreted with caution.

In the year immediately preceding diagnosis, YOAD patients had a larger morbidity burden than the cognitively healthy controls across multiple disease categories. This aligns with findings from our previous study,^10^ where we found increased healthcare utilization across all types of healthcare contacts in the year preceding YOAD diagnosis.

In examining the most frequent diagnoses among the study population, Symptoms involving cognitive functions and Other special examinations and investigations of persons without complaint had the highest IRRs for YOAD compared to controls. It is likely that these diagnostic codes were registered as part of the diagnostic evaluation leading up to referral or given at the initial memory clinic visit. In the sensitivity analysis censoring all diagnoses given 6 months before YOAD diagnosis, the disease categories containing these specific diagnostic codes were still significantly increased, perhaps signaling that diagnostic evaluation is often intricate and protracted due to the necessity for supplementary examinations. These diagnostic codes are rather unspecific though, perhaps implying that these patients have had symptoms for a long time without suspicion of dementia, supporting the need for aids in timely diagnosis.^19^, ^20^ Increased IRRs for diagnoses of injuries to the head and forearm suggests that YOAD patients may be more prone to injuries. In a large, register‐based study from Sweden, Nyström et al. assessed the association between injurious falls and Parkinson's disease up to 10 years prior to diagnosis and found an odds ratio of 1.19 (95% CI 1.08–1.31) in the time interval 7 to <10 years before diagnosis,^21^ which is comparable to IRRs found in the present study. Thus, injuries related to falls could potentially be an early symptom of neurodegeneration, even in younger patients.

The current study aimed at describing pre‐diagnostic symptoms of YOAD. While there is overlap between associations found in the present study and conditions previously described as risk factors for late‐onset dementia (such as hearing loss, hypertension, depression, etc.),^2^ our study did not aim to determine whether these conditions may also constitute a risk factor for YOAD (methods to tackle reverse causation and confounding would be needed). A large epidemiological study has suggested that patients with young onset all‐cause dementia have a larger vascular comorbidity burden than those without dementia.^22^ AD‐specific research shows that the prevalence of vascular risk factors is not elevated in YOAD patients, were apolipoprotein E (APOE) ε4 allele, that is, genetic risk factors, were instead more prominent.^23^ Future studies may examine the specific risk profile of YOAD, where APOE status would be relevant to include.

To our knowledge, no previous studies have examined the relationship between prior morbidity and YOAD. However, one study investigating morbidity prior to young onset all‐cause dementia found an increased odds ratio of dementia following anxiety, depression, diabetes, and stroke in the 2 years before dementia diagnosis.^24^ Similarly, a case‐control study of pre‐diagnostic symptoms in young onset all‐cause dementia found depression and anxiety among the most prevalent early symptoms.^7^ This is consistent with our results, where associations were also found for all corresponding disease categories.

Two other studies investigated morbidity preceding LOAD; a Taiwanese study of 4600 LOAD cases and 4600 controls found that anxiety, functional digestive disorder, psychopathology‐specific symptoms, disorders of the vestibular system, concussion, disorders of the urinary system, disorders of refraction and accommodation, and hearing loss were positively associated with LOAD.^25^ Likewise, a study based on the Baltimore Longitudinal Study of Aging found depression, erectile dysfunction, gait abnormalities, hearing loss, and nervous and musculoskeletal symptoms positively associated with LOAD.^26^ While some of these overlap with associations found in the present study (hearing loss, depression, disorders of the urinary system), others were not commonly found in our cohort (disorders of the vestibular system, disorders of refraction etc.).

Morbidity generally tends to increase with increasing age, and there is likely a difference in the morbidity pattern for those diagnosed with YOAD compared to LOAD based on age alone. Thus, morbidity in LOAD may differ considerably from morbidity in YOAD.

The association between psychiatric morbidity and dementia deserves particular attention. Prior studies have shown an association between mid‐ or late‐life depression and subsequent dementia in LOAD, suggesting that depression that begins in late‐life may be part of the AD prodrome.^1^, ^27^, ^28^, ^29^, ^30^ Results from the present study suggest this to be true for YOAD as well. Prior research has demonstrated a higher prevalence of depression in YOAD compared to LOAD,^31^ and it remains an area of great importance for clinicians to assess, both pre‐ and post‐diagnosis. Several studies have also shown an association between a diagnosis of stress in midlife and risk of dementia assuming stress as a risk factor for,^5^, ^32^ or early symptom in, dementia. In the present study, the association between psychiatric diagnoses and dementia was especially convincing immediately before the diagnosis of YOAD, suggesting that stress and depression may also be important early signs of YOAD.

Our study had several limitations. Inherent in the study design is the risk of detection bias; patients with comorbidities are more likely to see a medical doctor frequently, making them more likely to be referred to a memory clinic and receive a diagnosis upon experiencing cognitive symptoms than those who do not regularly see a doctor. However, 37% of cases were diagnosed in the moderate/severe stages of dementia, suggesting that frequent doctor visits do not guarantee a timely diagnosis. While censoring of contacts related to the diagnostic process in the memory clinic was attempted, it is not possible to completely remove all such contacts if not specifically marked with a dementia diagnosis. However, a sensitivity analysis removing all contacts 6 months prior to the index date yielded comparable results to the main analysis. Furthermore, as we did not have the date of symptom onset, we had to approximate this by using age at diagnosis. Another limitation of the present study is that the registers do not contain diagnostic information on morbidity from GPs. Patients with stress and depression are often seen by a GP or referred to a psychologist; therefore, we are likely to only register the more severe cases in the present study.

A major strength of the present study is the use of nationwide healthcare registers to assess morbidity registered at hospital contacts. In Denmark, hospital services are free of charge and the Danish healthcare registers contain detailed, reliable, and complete data on all hospital contacts. Thus, there is equal access to healthcare services for all and virtually full data coverage, limiting problems with selection bias. The use of DanDem for AD case finding ensures that all cases are diagnosed by specialists in memory clinics and allows access to detailed variables beyond the Danish registers. This allowed a novel approach to establish a large case cohort with a relatively rare disease.

Epidemiological studies on signs and symptoms in the prodromal phase of Parkinson's disease have helped to understand the course of the development of symptoms, and have changed the understanding of Parkinson's disease from a motor‐symptom disease to a disease of long latency, characterized by the progressive emergence of multiple non‐motor‐symptoms before onset of the typical motor‐symptoms.^33^ There is a similar potential for better understanding the disease course in YOAD, where epidemiological studies such as ours may help move toward a better understanding of the earliest disease stages. Especially the identification of psychiatric morbidity as a possible early sign of YOAD could potentially have an impact on screening instruments for early dementia, with more focus on behavioral and affective signs and symptoms along with cognitive assessment.

In conclusion, this study demonstrated a higher morbidity burden among YOAD patients than their matched controls, detectable up to as long as 10 years before dementia diagnosis. There was a substantial increase in psychiatric diagnoses, supporting prior research on stress and depression in the years leading up to diagnosis. Onset of psychiatric diseases or unspecific cognitive symptoms in midlife could potentially be an early warning sign of YOAD and may help guide GPs in which patients with cognitive complaints should be referred to a memory clinic. In our study, more than a third of all patients diagnosed with YOAD were diagnosed at an advanced stage in their disease, highlighting the need for tools to support a timely diagnosis. Future studies should further explore early symptoms and morbidity prior to a diagnosis of YOAD by exploring associations between reasons for GP contacts or use of prescription medication.

## CONFLICT OF INTEREST STATEMENT

All authors declare no conflicts of interest. Author disclosures are available in the supporting information.

## CONSENT STATEMENT

This research project was approved by the Danish Data Protection Agency, Statistics Denmark, and the Danish Health Data Authority. Danish law does not require ethics committee approval or informed patient consent.

## Supporting information

Supporting Information

Supporting Information

## Data Availability

All of the data used in this study are derived from the Danish National and Public Health registries. These data are collected and stored by the relevant authorities and cannot be made public or accessed by unauthorized parties. Access to such data is given via standard rules and regulations of data access outlined by the Danish Data Protection Agency and Danish Health Data Authority.
