# Supplementary material for: Mapping morbidity 10 years prior to a diagnosis of young onset Alzheimer's disease
Source: Alzheimers Dement. 2024 Jan 31;20(4):2373–83. doi: 10.1002/alz.13681 (PMC11032518; doi:10.1002/alz.13681)
Supplement: Supplementary file 1 — Supporting Information [file ALZ-20-2373-s001.pdf]

Supplementary tables and figures

Mapping morbidity 10 years prior to a diagnosis of young onset Alzheimer's disease

Damsgaard L, Janbek J, Laursen TM, Høgh P, Vestergaard K, Gottrup G, Jensen-Dahm C, Waldemar G. Alzheimer's & Dementia.

Correspondence: Line Damsgaard, MD

Danish Dementia Research Centre, Section 8008, Department of Neurology, Copenhagen University Hospital - Rigshospitalet, Copenhagen, Denmark

Email: line.damsgaard@regionh.dk

Contents:

Table S1. Censoring criteria

Table S2. Main analysis - Incidence rate ratios by disease category overall and in three time-intervals

Table S3. Sensitivity analysis censoring contacts 6 months before index date - Incidence rate ratios by disease category overall and in three time-intervals

Table S4. Most frequent diagnoses within each disease category and corresponding incidence rate ratios

Table S5. Sensitivity analysis by dementia syndrome severity at time of diagnosis - Incidence rate ratios by disease category overall and in three time-intervals

Table S6. Post-hoc analysis - Incidence rate ratios for psychiatric subcategories

Table S1. Censoring criteria

	Cases	Controls
**Developmental disorders and mental retardation**	X	X
ICD-8: 311-315, 759.3, ICD-10: DF70-DF79, DQ90		
**Not living in Denmark in 10-year retrospective period**	X	X
**Dementia or mild cognitive impairment diagnosis**	*	X
ICD-8: 290.09-11, 290.18-19, 293.09-19, ICD 10: F00.0-00.9, F01.0-01.9, F02.0-F02.8, F03.9, F04.0-F04.9, F06.7 G30.0-G30.9, G31.0A, G31.0B, G31.8, G31.8E, G31.9		
**Dementia medication**		X
ATC code N06DA02-4, N06DX01		
**Entry in DanDem**		X

***** ICD-codes registered in the Danish National Patient Register are censored in the conditional logistic regression and frequency analyses. This does not impact case selection.

ICD: International classification of diseases, ATC: Anatomical therapeutic chemical code, DanDem: Danish Quality Database for Dementia

Table S2. Incidence rate ratios by disease category overall and in three time-intervals

ICD-10 code range and chapters		Time-period	Unadjusted		Adjusted	
			IRR	95% CI	IRR	95% CI
**A00-B99**	**I**	**Overall**	**1.01**	**0.82-1.25**	**1.01**	**0.82-1.24**
Certain infections		10->5 years prior to index date	0.80	0.56-1.27	0.81	0.57-1.14
		5->1 years prior to index date	1.13	0.86-1.49	1.13	0.86-1.48
		≤1 year prior to index date	1.29	0.83-2.01	1.24	0.79-1.93
**C00-D48**	**II**	**Overall**	**1.03**	**0.90-1.18**	**1.03**	**0.90-1.17**
Neoplasms		10->5 years prior to index date	0.92	0.76-1.11	0.92	0.76-1.11
		5->1 years prior to index date	1.01	0.85-1.19	1.00	0.85-1.19
		≤1 year prior to index date	1.00	0.79-1.26	0.98	0.78-1.25
**D50-D89**	**III**	**Overall**	**1.26**	**0.94-1.68**	**1.22**	**0.91-1.63**
Hematological/immunological diseases		10->5 years prior to index date	1.42	0.93-2.17	1.41	0.92-2.15
		5->1 years prior to index date	1.14	0.75-1.74	1.09	0.71-1.66
		≤1 year prior to index date	1.74	1.03-2.94	1.71	1.01-2.89
**E00-E90**	**IV**	**Overall**	**1.38**	**3.22-1.57**	**1.38**	**1.21-1.57**
Endocrine/metabolic diseases		10->5 years prior to index date	1.11	0.94-1.31	1.11	0.93-1.31
		5->1 years prior to index date	1.07	0.91-1.26	1.06	0.90-1.25
		≤1 year prior to index date	2.66	2.19-3.24	2.64	2.17-3.21
**F00-F99**	**V**	**Overall**	**3.14**	**2.70-3.65**	**3.18**	**2.73-3.71**
Mental and behavioral disorders*		10->5 years prior to index date	1.45	1.16-1.81	1.43	1.14-1.79
		5->1 years prior to index date	2.52	2.06-3.08	2.48	2.02-3.04
		≤1 year prior to index date	8.07	6.19-10.52	8.19	6.27-10.70
**G00-G99**	**VI**	**Overall**	**1.52**	**1.31-1.76**	**1.52**	**1.31-1.77**
Diseases of the nervous system*		10->5 years prior to index date	1.00	0.80-1.24	1.01	0.81-1.26
		5->1 years prior to index date	1.06	0.86-1.30	1.05	0.86-1.30
		≤1 year prior to index date	3.16	2.48-4.02	3.16	2.48-4.03
**H00-H59**	**VII**	**Overall**	**1.20**	**1.03-1.41**	**1.20**	**1.03-1.41**
Diseases of the eye and adnexa		10->5 years prior to index date	1.02	0.81-1.29	1.03	0.81-1.30
		5->1 years prior to index date	1.17	0.96-1.43	1.17	0.96-1.43
		≤1 year prior to index date	1.47	1.10-1.96	1.45	1.08-1.93
**H60-H95**	**VIII**	**Overall**	**1.44**	**1.20-1.73**	**1.45**	**1.20-1.74**
Diseases of the ear and mastoid process		10->5 years prior to index date	1.51	1.16-1.97	1.51	1.16-1.97
		5->1 years prior to index date	1.43	1.14-1.80	1.45	1.15-1.81
		≤1 year prior to index date	1.40	1.00-1.96	1.41	1.01-1.98
**I00-I99**	**IX**	**Overall**	**1.25**	**1.12-1.41**	**1.26**	**1.12-1.41**
Diseases of the circulatory system		10->5 years prior to index date	1.05	0.90-1.22	1.05	0.90-1.22
		5->1 years prior to index date	1.08	0.94-1.25	1.08	0.94-1.25
		≤1 year prior to index date	2.14	1.81-2.53	2.14	1.81-2.53

ICD-10 code range and chapters		Time-period	Unadjusted		Adjusted	
			IRR	95% CI	IRR	95% CI
**J00-J99**	**X**	**Overall**	**1.14**	**0.97-1.34**	**1.12**	**0.95-1.31**
Diseases of the respiratory system		10->5 years prior to index date	1.02	0.81-1.29	1.01	0.80-1.28
		5->1 years prior to index date	1.05	0.85-1.30	1.01	0.82-1.26
		≤1 year prior to index date	1.42	1.09-1.87	1.38	1.05-1.81
**K00-K93**	**XI**	**Overall**	**1.01**	**0.90-1.15**	**1.01**	**0.89-1.14**
Diseases of the digestive system		10->5 years prior to index date	1.05	0.89-1.21	1.05	0.89-1.23
		5->1 years prior to index date	0.97	0.83-1.13	0.97	0.83-1.13
		≤1 year prior to index date	0.99	0.79-1.26	0.98	0.78-1.24
**L00-L99**	**XII**	**Overall**	**1.17**	**0.97-1.42**	**1.17**	**0.97-1.42**
Diseases of the skin/subcutaneous system		10->5 years prior to index date	1.12	0.85-1.47	1.12	0.85-1.48
		5->1 years prior to index date	1.06	0.82-1.37	1.06	0.82-1.36
		≤1 year prior to index date	2.00	1.37-2.93	2.00	1.37-2.94
**M00-M99**	**XIII**	**Overall**	**0.92**	**0.83-1.03**	**0.92**	**0.83-1.03**
Diseases of the musculoskeletal system		10->5 years prior to index date	1.00	0.88-1.34	1.00	0.88-1.14
		5->1 years prior to index date	0.83	0.73-0.95	0.83	0.73-0.95
		≤1 year prior to index date	1.02	0.85-1.22	1.02	0.85-1.22
**N00-N99**	**XIV**	**Overall**	**1.11**	**0.97-1.27**	**1.10**	**0.96-1.26**
Diseases of the genitourinary system		10->5 years prior to index date	1.06	0.89-1.27	1.06	0.89-1.26
		5->1 years prior to index date	1.08	0.91-1.29	1.08	0.90-1.28
		≤1 year prior to index date	1.26	0.96-1.65	1.24	0.94-1.62
**R00-R99**	**XVIII**	**Overall**	**2.46**	**2.20-2.76**	**2.45**	**2.19-2.74**
Symptoms/signs not classified elsewhere		10->5 years prior to index date	1.19	1.04-1.37	1.19	1.03-1.36
		5->1 years prior to index date	1.41	1.25-1-59	1.40	1.24-1.59
		≤1 year prior to index date	5.73	4.93-6.66	5.69	4.89-6.61
**S00-T98**	**XIX**	**Overall**	**1.32**	**1.81-1.47**	**1.31**	**1.17-1.46**
Injuries, poisoning, and other external causes		10->5 years prior to index date	1.14	1.01-1.28	1.13	1.00-1.27
		5->1 years prior to index date	1.36	1.21-1.54	1.35	1.20-1.53
		≤1 year prior to index date	1.40	1.17-1.67	1.39	1.16-1.66
**Z00-Z80**	**XXI**	**Overall**	**8.50**	**5.56-12.99**	**8.80**	**5.74-13.48**
Factors influencing health status etc.		10->5 years prior to index date	1.07	0.93-1.24	1.07	0.93-1.25
		5->1 years prior to index date	1.61	3.37-1.90	1.63	1.39-1.92
		≤1 year prior to index date	6.90	5.92-8.04	7.01	6.01-8.17

Incidence rate ratios (IRRs) for young onset Alzheimer's disease are presented overall and in 3 time-intervals prior to diagnosis with 95% confidence intervals (CI). The adjusted IRRs are adjusted for age, sex, highest attained educational level at age 40 years (or at time of diagnosis if age at diagnosis <40 years), and civil status at index date. ICD: International classification of diseases

* Excluding mild cognitive impairment and dementia diagnoses

Table S3. Sensitivity analysis censoring contacts 6 months before index date - Incidence rate ratios by disease category overall and in three time-intervals

ICD-10 code range and chapters	Time-period	Unadjusted		Adjusted	
		IRR	95% CI	IRR	95% CI
**A00-B99 I**	**Overall**	0.99	0.79-1.23	0.98	0.79-1.22
Certain infections	10->5 years prior to index date	0.80	0.56-1.13	0.81	0.57-1.14
	5->1 years prior to index date	1.13	0.86-1.49	1.13	0.86-1.48
	≤1 year-6 months prior to index date	1.09	0.53-2.11	1.04	0.54-2.02
**C00-D48 II**	**Overall**	1.03	0.90-1.18	1.03	0.90-1.18
Neoplasms	10->5 years prior to index date	0.92	0.76-1.11	0.92	0.76-1.11
	5->1 years prior to index date	1.00	0.85-1.19	1.00	0.85-1.19
	≤1 year-6 months prior to index date	1.04	0.77-1.41	1.03	0.76-1.40
**D50-D89 III**	**Overall**	1.21	0.89-1.65	1.17	0.86-1.60
Hematological/immunological diseases	10->5 years prior to index date	1.42	0.93-2.17	1.41	0.92-2.15
	5->1 years prior to index date	1.14	0.75-1.74	1.09	0.71-1.66
	≤1 year-6 months prior to index date	1.83	0.87-3.88	1.84	0.87-3.90
**E00-E90 IV**	**Overall**	1.18	1.04-1.35	1.18	1.03-1.35
Endocrine/metabolic diseases	10->5 years prior to index date	1.11	0.94-1.31	1.11	0.93-1.31
	5->1 years prior to index date	1.07	0.91-1.26	1.06	0.90-1.25
	≤1 year-6 months prior to index date	1.81	1.38-2.38	1.79	1.36-2.35
**F00-F99 V**	**Overall**	2.47	2.11-2.90	2.48	2.11-2.91
Mental and behavioral disorders*	10->5 years prior to index date	1.45	1.16-1.81	1.43	1.14-1.79
	5->1 years prior to index date	2.52	2.06-3.08	2.48	2.02-3.04
	≤1 year-6 months prior to index date	5.10	3.59-7.26	5.14	3.60-7.32
**G00-G99 VI**	**Overall**	1.19	1.01-1.40	1.19	1.02-1.40
Diseases of the nervous system*	10->5 years prior to index date	1.00	0.80-1.24	1.01	0.81-1.26
	5->1 years prior to index date	1.06	0.86-1.30	1.05	0.86-1.30
	≤1 year-6 months prior to index date	2.12	1.49-3.01	2.13	1.50-3.03
**H00-H59 VII**	**Overall**	1.13	0.96-1.33	1.13	0.96-1.33
Diseases of the eye and adnexa	10->5 years prior to index date	1.02	0.81-1.29	1.03	0.81-1.30
	5->1 years prior to index date	1.17	0.96-1.43	1.17	0.96-1.43
	≤1 year-6 months prior to index date	0.79	0.51-1.24	0.79	0.50-1.24
**H60-H95 VIII**	**Overall**	1.39	1.15-1-68	1.40	1.16-1.69
Diseases of the ear and mastoid process	10->5 years prior to index date	1.51	1.16-1.97	1.51	1.16-1.97
	5->1 years prior to index date	1.43	1.14-1.80	1.45	1.15-1.81
	≤1 year-6 months prior to index date	0.86	0.52-1.44	0.89	0.54-1.49
**I00-I99 IX**	**Overall**	1.10	0.97-1.24	1.10	0.97-1.24
Diseases of the circulatory system	10->5 years prior to index date	1.05	0.90-1.22	1.05	0.90-1.22
	5->1 years prior to index date	1.08	0.94-1.25	1.08	0.94-1.25
	≤1 year-6 months prior to index date	1.63	1.29-2.06	1.63	1.29-2.06

ICD-10 code range and chapters	Time-period	Unadjusted		Adjusted	
		IRR	95% CI	IRR	95% CI
**J00-J99 X**	**Overall**	1.13	0.96-1.33	1.11	0.94-1.31
Diseases of the respiratory system	10->5 years prior to index date	1.02	0.81-1.29	1.01	0.80-1.28
	5->1 years prior to index date	1.05	0.85-1.30	1.01	0.82-1.26
	≤1 year-6 months prior to index date	1.37	0.95-1.97	1.33	0.92-1.91
**K00-K93 XI**	**Overall**	1.03	0.91-1-17	1.03	0.90-1.16
Diseases of the digestive system	10->5 years prior to index date	1.05	0.90-1.23	1.05	0.89-1.23
	5->1 years prior to index date	0.97	0.83-1.13	0.97	0.83-1.13
	≤1 year-6 months prior to index date	1.03	0.76-1.40	1.01	0.74-1.38
**L00-L99 XII**	**Overall**	1.11	0.92-1.35	1.12	0.92-1.36
Diseases of the skin/subcutaneous	10->5 years prior to index date	1.12	0.85-1.47	1.12	0.85-1.48
system	5->1 years prior to index date	1.06	0.82-1.37	1.06	0.82-1.36
	≤1 year-6 months prior to index date	2.46	1.48-4.08	2.49	1.50-4.15
**M00-M99 XIII**	**Overall**	0.90	0.80-1.00	0.90	0.80-1.00
Diseases of the musculoskeletal	10->5 years prior to index date	1.00	0.88-1.14	1.00	0.88-1.14
system	5->1 years prior to index date	0.83	0.73-0.95	0.83	0.73-0.95
	≤1 year-6 months prior to index date	0.94	0.73-1.20	0.94	0.73-1.20
**N00-N99 XIV**	**Overall**	1.10	0.96-1.26	1.09	0.95-1.25
Diseases of the genitourinary system	10->5 years prior to index date	1.06	0.89-1.27	1.06	0.89-1.26
	5->1 years prior to index date	1.08	0.91-1.29	1.08	0.90-1.28
	≤1 year-6 months prior to index date	1.37	0.96-1.96	1.32	0.92-1.89
**R00-R99 XVIII**	**Overall**	1.50	1.35-1.68	1.49	1.34-1.67
Symptoms/signs not classified	10->5 years prior to index date	1.19	1.04-1.37	1.19	1.03-1.36
elsewhere	5->1 years prior to index date	1.41	1.25-1.59	1.40	1.24-1.59
	≤1 year-6 months prior to index date	2.15	1.75-2.64	2.13	1.73-2.62
**S00-T98 XIX**	**Overall**	1.31	1.17-1.46	1.30	1.16-1.45
Injuries, poisoning, and other	10->5 years prior to index date	1.14	1.01-1.28	1.13	1.00-1.27
external causes	5->1 years prior to index date	1.36	1.21-1.54	1.35	1.20-1.53
	≤1 year-6 months prior to index date	1.29	1.01-1.65	1.29	1.01-1.66
**Z00-Z80 XXI**	**Overall**	1.95	1.56-2.44	1.99	1.59-2.49
Factors influencing health status etc	10->5 years prior to index date	1.07	0.93-1.24	1.07	0.93-1.25
	5->1 years prior to index date	1.61	1.37-1.90	1.63	1.39-1.92
	≤1 year-6 months prior to index date	2.16	1.92-2.42	2.17	1.93-2.43

Incidence rate ratios (IRRs) for young onset Alzheimer's disease cases are presented overall and in 3 time-intervals prior to diagnosis with 95% confidence intervals (CI) after censoring all contacts in the 6 months prior to index date. The adjusted IRRs are adjusted for age, sex, highest attained educational level at age 40 years (or at time of diagnosis if age at diagnosis <40 years), and civil status at index date. ICD: International classification of diseases

* Excluding mild cognitive impairment and dementia diagnoses

Table S4. Most frequent diagnoses within each disease category and corresponding incidence rate ratios

ICD-10 code range and chapters	Most frequent diagnoses	n	Unadjusted		Adjusted	
			IRR	95% CI	IRR	95% CI
**A00-B99 I**						
Certain infections	A46: Erysipelas	111	1.06	0.69-1.63	1.06	0.69-1.63
	A41: Other sepsis	95	1.01	0.64-1.61	1.00	0.63-1.59
	A49: Bacterial infection of unspecified site	58	1.46	0.85-2.53	1.45	0.84-2.51
**C00-D48 II**						
Neoplasms	D12: Benign neoplasm of colon etc.	391	1.22	0.98-1.53	1.23	0.98-1.54
	C50: Malignant neoplasm of breast	172	1.07	0.75-1.51	1.06	0.74-1.49
	D17: Benign lipomatous neoplasm	110	0.83	0.52-1.30	0.83	0.53-1.31
**D50-D89 III**						
Hematological/immunological diseases	D64: Other anaemias	82	1.32	0.82-2.12	1.26	0.78-2.03
	D50: Iron deficiency anaemia	47	1.41	0.76-2.60	1.36	0.73-2.52
	D68: Other coagulation defects	22	0.30	0.07-1.28	0.30	0.07-1.29
**E00-E90 IV**						
Endocrine/metabolic diseases	E78: Disorders of lipoprotein metabolism etc.	498	1.63	1.34-1.97	1.64	1.35-1.99
	E11: Type 2 diabetes mellitus	401	1.20	0.96-1.50	1.20	0.96-1.51
	E66: Obesity	280	0.59	0.43-0.81	0.59	0.43-0.81
**F00-F99 V**						
Mental and behavioral disorders*	F10: Alcohol-related mental disorders	225	2.12	1.62-2.79	2.12	1.62-2.79
	F43: Reaction to severe stress	191	2.82	2.11-3.78	2.77	2.06-3.70
	F32: Depressive episode	159	3.57	2.60-4.89	3.48	2.54-4.77
**G00-G99 VI**						
Diseases of the nervous system*	G56: Mononeuropathies of arm	192	0.73	0.51-1.04	0.73	0.51-1.05
	G47: Sleep disorders	163	1.01	0.71-1.44	1.02	0.71-1.45
	G45: Transient cerebral ischaemic attacks	114	1.51	1.02-2.23	1.56	1.05-2.31
**H00-H59 VII**						
Diseases of the eye and adnexa	H25: Senile cataract	301	1.67	1.31-2.12	1.66	1.31-2.12
	H26: Other cataract	94	1.03	0.64-1.65	1.03	0.77-1.65
	H35: Other retinal disorders	94	0.56	0.32-0.98	0.56	0.32-0.97
**H60-H95 VIII**						
Diseases of the ear and mastoid process	H91: Other hearing loss	362	1.50	1.19-1.88	1.51	1.20-1.90
	H93: Other disorders of ear	161	1.28	0.91-1.80	1.30	0.92-1.83
	H90: Conductive and sensorineural hearing loss	126	1.34	0.91-1.96	1.33	0.91-1.95
**I00-I99 IX**						
Diseases of the circulatory system	I10: Essential hypertension	1024	1.39	1.20-1.60	1.38	1.19-1.60
	I20: Angina pectoris	309	1.22	0.95-1.57	1.23	0.95-1.58
	I25: Chronic ischaemic heart disease	298	1.11	0.85-1.44	1.11	0.85-1.44

ICD-10 code range and chapters	Most frequent diagnoses	n	Unadjusted		Adjusted	
			IRR	95% CI	IRR	95% CI
**J00-J99 X**						
Diseases of the respiratory system	J44: Chronic obstructive pulmonary disease	249	1.09	0.82-1.45	1.06	0.79-1.41
	J18: Pneumonia, organism unspecified	243	1.01	0.82-1.48	1.07	0.80-1.43
	J45: Asthma	169	1.09	0.77-1.54	1.09	0.77-1.54
**K00-K93 XI**						
Diseases of the digestive system	K57: Diverticular disease of intestine	284	1.09	0.83-1.42	1.09	0.84-1.43
	K59: Other intestinal disorders	229	1.34	1.00-1.78	1.33	1.00-1.77
	K40: Inguinal hernia	216	0.99	0.72-1.37	0.99	0.72-1.37
**L00-L99 XII**						
Diseases of the skin/subcutaneous system	L02: Cutaneous abscess etc.	100	1.17	0.75-1.82	1.19	0.77-1.85
	L08: Other local infections of skin	71	1.45	0.88-2.40	1.43	0.87-2.37
	L40: Psoriasis	58	1.24	0.71-2.19	1.24	0.70-2.19
**M00-M99 XIII**						
Diseases of the musculoskeletal system	M17: Gonarthrosis	495	1.08	0.88-1.33	1.08	0.88-1.33
	M75: Shoulder lesions	481	0.91	0.74-1.14	0.92	0.74-1.14
	M23: Internal derangement of knee	349	0.96	0.75-1.23	0.96	0.75-1.24
**N00-N99 XIV**						
Diseases of the genitourinary system	N39: Other disorders of urinary system	235	1.36	1.03-1.80	1.34	1.01-1.78
	N30: Cystitis	190	1.41	1.03-1.92	1.39	1.02-1.91
	N81: Female genital prolapse	163	1.00	0.70-1.43	0.99	0.69-1.42
**R00-R99 XVIII**						
Symptoms/signs not classified elsewhere	R10: Abdominal and pelvic pain	576	1.04	0.85-1.26	1.03	0.85-1.25
	R41: Symptoms involving cognitive functions	415	90.86	52.29-157.86	90.84	52.27-157.87
	R07: Pain in throat and chest	302	1.27	0.99-1.64	1.27	0.98-1.64
**S00-T98 XIX**						
Injuries, poisoning, and other external causes	S61: Open wound of wrist and hand	486	1.25	1.02-1.54	1.25	1.02-1.53
	S52: Fracture of forearm	338	1.47	1.16-1.86	1.45	1.15-1.84
	S01: Open wound of head	280	1.56	1.21-2.01	1.55	1.20-1.99
**Z00-Z80 XXI**						
Factors influencing health status etc	Z01: Investigations without complaints	6039	4.53	3.54-5.79	4.65	3.63-5.96
	Z03: Observation	3338	2.60	2.32-2.91	2.59	2.31-2.91
	Z12: Screening for neoplasms	1643	0.91	0.78-1-06	0.92	0.78-1.06

Incidence rate ratios (IRRs) for young onset Alzheimer's disease for the three most frequent diagnoses in the study population in each disease category with 95% confidence intervals (CI). The adjusted IRRs are adjusted for age, sex, highest attained educational level at age 40 years (or at time of diagnosis if age at diagnosis <40 years), and civil status at index date. ICD: International classification of diseases

* Excluding mild cognitive impairment and dementia diagnoses

Table S5. Sensitivity analysis by dementia syndrome severity at time of diagnosis - Incidence rate ratios by disease category overall and in three time-intervals

ICD-10 code range and chapters	Time-period	MCI/MILD DEMENTIA				MODERATE/SEVERE DEMENTIA			
		Unadjusted		Adjusted		Unadjusted		Adjusted	
		IRR	95% CI	IRR	95% CI	IRR	95% CI	IRR	95% CI
**A00-B99 I**	**Overall**	**0.95**	**0.72-1.24**	**0.97**	**0.74-1.27**	**1.13**	**0.91-1.56**	**1.07**	**0.76-1.51**
	10->5 years prior to index date	0.93	0.61-1.40	0.96	0.64-1.45	0.58	0.30-1.10	0.59	0.31-1.13
	5->1 years prior to index date	0.97	0.68-1.38	0.98	0.68-1.41	1.43	0.94-2.19	1.37	0.89-2.10
	≤1 year prior to index date	0.79	0.41-1.53	0.84	0.43-1.63	2.22	1.19-4.15	1.89	1.00-3.60
**C00-D48 II**	**Overall**	**1.01**	**0.85-1.19**	**1.00**	**0.84-1.19**	**1.07**	**0.86-1.32**	**1.09**	**0.88-1.35**
Neoplasms	10->5 years prior to index date	0.93	0.74-1.18	0.94	0.74-1.19	0.89	0.66-1.20	0.92	0.68-1.25
	5->1 years prior to index date	0.94	0.75-1.67	0.94	0.75-1.67	1.12	0.86-1.47	1.14	0.87-1.49
	≤1 year prior to index date	0.87	0.63-1.19	0.88	0.64-1.20	1.21	0.85-1.72	1.20	0.84-1.72
**D50-D89 III**	**Overall**	**0.97**	**0.65-1.44**	**1.00**	**0.67-1.50**	**1.78**	**1.15-2.75**	**1.63**	**1.05-2.53**
Hematological/immunological diseases	10->5 years prior to index date	1.10	0.60-2.00	1.10	0.61-2.01	1.89	1.03-3.47	1.82	0.98-3.37
	5->1 years prior to index date	0.94	0.54-1.65	0.98	0.55-1.72	1.51	0.79-2.89	1.36	0.70-2.64
	≤1 year prior to index date	1.13	0.52-2.42	1.18	0.55-2.54	2.79	1.31-5.93	2.65	1.24-5.69
**E00-E90 IV**	**Overall**	**1.27**	**1.08-1.50**	**1.31**	**1.11-1.54**	**1.58**	**1.29-1.94**	**1.51**	**1.22-1.86**
Endocrine/metabolic diseases	10->5 years prior to index date	1.04	0.84-1.29	1.07	0.86-1.32	1.23	0.93-1.61	1.19	0.90-1.57
	5->1 years prior to index date	0.97	0.79-1.20	1.00	0.81-1.23	1.24	0.96-1.60	1.18	0.91-1.53
	≤1 year prior to index date	2.61	2.03-3.35	2.68	2.08-3.45	2.74	2.00-3.75	2.55	1.85-3.51
**F00-F99 V**	**Overall**	**2.39**	**1.97-2.91**	**2.56**	**2.10-3.12**	**4.78**	**3.73-6.11**	**4.47**	**3.48-5.75**
Mental and behavioral disorders*	10->5 years prior to index date	1.16	0.86-1.56	1.20	0.89-1.63	1.98	1.42-2.77	1.81	1.28-2.55
	5->1 years prior to index date	1.87	1.44-2.45	2.01	1.53-2.63	3.82	2.78-5.24	3.41	2.47-4.71
	≤1 year prior to index date	6.08	4.39-8.42	6.43	4.63-8.94	13.20	8.23-21.19	15.56	7.77-20.33
**G00-G99 VI**	**Overall**	**1.35**	**1.12-1.63**	**1.38**	**1.14-1.67**	**1.85**	**1.45-2.35**	**1.85**	**1.45-2.36**
	10->5 years prior to index date	0.83	0.62-1.10	0.84	0.63-1.12	1.39	0.97-1.98	1.42	0.99-2.04
	5->1 years prior to index date	0.91	0.69-1.19	0.93	0.71-1.22	1.33	0.97-1.83	1.33	0.96-1.84
	≤1 year prior to index date	3.06	2.26-4.16	3.10	2.28-4.21	3.32	2.24-4.91	3.33	2.23-4.96
**H00-H59 VII**	**Overall**	**1.18**	**0.97-1.43**	**1.18**	**0.97-1.44**	**1.25**	**0.97-1.62**	**1.25**	**0.97-1.56**
Diseases of the eye and adnexa	10->5 years prior to index date	1.01	0.76-1.35	1.02	0.76-1.36	1.04	0.70-1.54	1.02	0.69-1.52
	5->1 years prior to index date	1.15	0.90-1.48	1.16	0.90-1.49	1.20	0.85-1.70	1.24	0.87-1.76
	≤1 year prior to index date	1.25	0.86-1.82	1.25	0.86-1.83	1.88	1.20-2.96	1.82	1.15-2.89
**H60-H95 VIII**	**Overall**	**1.47**	**1.67-1.85**	**1.47**	**1.17-1-86**	**1.38**	**1.02-1.88**	**1.42**	**1.04-1.94**
Diseases of the ear and mastoid process	10->5 years prior to index date	1.50	1.08-2.10	1.48	1.06-2.08	1.53	1.00-2.33	1.49	0.97-2.29
	5->1 years prior to index date	1.47	1.12-1.95	1.48	1.12-1.96	1.35	0.92-2.00	1.40	0.94-2.08
	≤1 year prior to index date	1.58	1.04-2.40	1.55	1.02-2.36	1.12	0.63-2.00	1.17	0.65-2.12
**I00-I99 IX**	**Overall**	**1.24**	**1.07-1.43**	**1.26**	**1.09-1.46**	**1.28**	**1.06-1.56**	**1.28**	**1.05-1.55**
Diseases of the circulatory system	10->5 years prior to index date	1.00	0.82-1.21	1.00	0.83-1.22	1.14	0.90-1.45	1.13	0.88-1.44
	5->1 years prior to index date	1.07	0.90-1.28	1.09	0.91-1.30	1.10	0.87-1.38	1.10	0.87-1.39
	≤1 year prior to index date	2.05	1.66-2.54	2.06	1.67-2.55	2.30	1.75-3.02	2.28	1.73-3.01

ICD-10 code range and chapters	Time-period	MCI/MILD DEMENTIA				MODERATE/SEVERE DEMENTIA			
		Unadjusted		Adjusted		Unadjusted		Adjusted	
		IRR	95% CI	IRR	95% CI	IRR	95% CI	IRR	95% CI
**J00-J99 X**	**Overall**	**1.12**	**0.91-1.36**	**1.14**	**0.93-1.39**	**1.18**	**0.91-1.53**	**1.12**	**0.86-1.46**
Diseases of the respiratory system	10->5 years prior to index date	0.92	0.68-1.23	0.93	0.69-1.25	1.23	0.85-1.77	1.20	0.83-1.74
	5->1 years prior to index date	1.02	0.78-1.34	1.04	0.80-1.37	1.09	0.77-1.53	1.01	0.71-1.42
	≤1 year prior to index date	1.53	1.10-2.13	1.56	1.12-2.17	1.25	0.78-1.98	1.13	0.70-1.82
**K00-K93 XI**	**Overall**	**0.91**	**0.78-1.07**	**0.92**	**0.78-1.08**	**1.19**	**0.98-1.46**	**1.18**	**0.96-1.44**
Diseases of the digestive system	10->5 years prior to index date	1.04	0.84-1.27	1.05	0.85-1.29	1.06	0.81-1.39	1.03	0.78-1.35
	5->1 years prior to index date	0.86	0.71-1.05	0.87	0.71-1.05	1.17	0.92-1.50	1.16	0.91-1.49
	≤1 year prior to index date	0.67	0.47-0.94	0.67	0.48-0.95	1.55	1.11-2.16	1.51	1.08-2.11
**L00-L99 XII**	**Overall**	**1.07**	**0.84-1.36**	**1.08**	**0.85-1.37**	**1.37**	**1.01-1.85**	**1.32**	**0.97-1.79**
Diseases of the skin/subcutaneous system	10->5 years prior to index date	1.10	0.78-1.56	1.10	0.78-1.56	1.15	0.73-1.80	1.10	0.70-1.74
	5->1 years prior to index date	0.96	0.70-1.33	0.96	0.70-1.33	1.26	0.83-1.92	1.24	0.81-1.90
	≤1 year prior to index date	1.26	0.72-2.18	1.27	0.73-2.20	3.39	1.94-5.94	3.21	1.82-5.67
**M00-M99 XIII**	**Overall**	**1.02**	**0.88-1.17**	**1.02**	**0.88-1.17**	**0.79**	**0.65-0.95**	**0.79**	**0.66-0.96**
Diseases of the musculoskeletal system	10->5 years prior to index date	1.08	0.92-1.26	1.09	0.93-1.28	0.89	0.72-1.10	0.88	0.71-1.09
	5->1 years prior to index date	0.90	0.77-1.06	0.91	0.77-1.07	0.73	0.59-0.90	0.73	0.59-0.91
	≤1 year prior to index date	1.10	0.88-1.38	1..11	0.88-1.40	0.90	0.67-1.22	0.92	0.68-1.24
**N00-N99 XIV**	**Overall**	**1.18**	**1.00-1.39**	**1.19**	**1.01-1.41**	**1.00**	**0.80-1.25**	**1.00**	**0.80-1.25**
Diseases of the genitourinary system	10->5 years prior to index date	1.09	0.88-1.36	1.09	0.87-1.36	1.01	0.76-1.36	1.02	0.76-1.37
	5->1 years prior to index date	1.19	0.96-1.48	1.22	0.98-1.51	0.91	0.67-1.22	0.88	0.65-1.19
	≤1 year prior to index date	1.11	0.77-1.61	1.16	0.80-1.68	1.47	0.99-2.18	1.44	0.97-2.15
**R00-R99 XVIII**	**Overall**	**2.31**	**2.01-2.66**	**2.32**	**2.01-2.67**	**2.76**	**2.29-3.23**	**2.73**	**2.26-3.30**
Symptoms/signs not classified elsewhere	10->5 years prior to index date	1.14	0.96-1.36	1.15	0.96-1.37	1.28	1.02-1.61	1.26	1.00-1.58
	5->1 years prior to index date	1.41	1.20-1.65	1.42	1.21-1.66	1.41	1.16-1.72	1.39	1.14-1.70
	≤1 year prior to index date	5.54	4.58-6.69	5.55	4.59-6.72	6.08	4.74-7.78	6.00	4.67-7.71
**S00-T98 XIX**	**Overall**	**1.34**	**1.17-1.54**	**1.34**	**1.17-1.54**	**1.28**	**1.07-1.53**	**1.25**	**1.04-1.49**
Injuries, poisoning, and other external causes	10->5 years prior to index date	1.17	1.01-1.36	1.18	1.01-1.37	1.08	0.90-1.32	1.05	0.86-1.28
	5->1 years prior to index date	1.35	1.16-1.58	1.36	1.16-1.59	1.37	1.12-1.68	1.34	1.10-1.64
	≤1 year prior to index date	1.26	1.00-1.58	1.28	1.01-1.61	1.66	1.24-2.22	1.62	1.20-2.18
**Z00-Z80 XXI**	**Overall**	**7.29**	**4.50-11.82**	**7.53**	**4.63-12.22**	**12.82**	**5.22-31.52**	**13.82**	**5.59-34.13**
Factors influencing health status etc.	10->5 years prior to index date	0.99	0.82-1.18	0.99	0.83-1.18	1.27	0.98-1.64	1.27	0.98-1.65
	5->1 years prior to index date	1.68	1.38-2.06	1.70	1.39-2.08	1.49	1.14-1.96	1.55	1.17-2.05
	≤1 year prior to index date	8.76	7.11-10.79	9.03	7.31-11.15	4.95	3.95-6.21	5.21	4.13-6.57

Incidence rate ratios (IRRs) for young onset Alzheimer's disease cases are presented overall and in 3 time-intervals prior to diagnosis with 95% confidence intervals (CI) according to dementia syndrome severity at time of diagnosis (patients with mild cognitive impairment (MCI) or mild dementia compared to their controls, and patients with moderate/severe dementia compared to their controls). The adjusted IRRs are adjusted for age, sex, highest attained educational level at age 40 years (or at time of diagnosis if age at diagnosis <40 years), and civil status at index date. ICD: International classification of diseases

* Excluding mild cognitive impairment and dementia diagnoses

Table S6. Post-hoc analysis - Incidence rate ratios for psychiatric subcategories

ICD-10 code range and chapters	Time-period	Unadjusted		Adjusted	
		IRR	95% CI	IRR	95% CI
**DF00-09**	**Overall**	8.37	4.81-14.55	8.05	4.62-14.03
Organic, including symptomatic,	10->5 years prior to index date	3.00	0.87-10.36	2.99	0.86-10.33
mental disorders*	5->1 years prior to index date	4.15	2.04-8.48	3.88	1.89-7.96
	≤1 year prior to index date	27.00	8.19-89.00	26.39	8.00-87.03
**DF10-19**	**Overall**	1.84	1.46-2.33	1.81	1.43-2.29
Mental and behavioral disorders due to	10->5 years prior to index date	1.23	0.87-1.74	1.20	0.85-1.71
psychoactive substance use	5->1 years prior to index date	1.40	0.98-1.99	1.33	0.93-1.91
	≤1 year prior to index date	4.62	3.07-6.94	4.57	3.03-6.88
**DF20-29**	**Overall**	2.25	1.45-3.50	2.28	1.46-3.57
Schizophrenia, schizotypal and	10->5 years prior to index date	0.95	0.49-1.82	0.95	0.49-1.83
delusional disorders	5->1 years prior to index date	1.84	1.00-3.38	1.88	1.02-3.47
	≤1 year prior to index date	5.25	2.58-10.67	5.37	2.62-11.01
**DF30-39**	**Overall**	3.20	2.53-4.04	3.14	2.48-3.98
Mood/affective disorders	10->5 years prior to index date	1.50	1.05-2.14	1.45	1.01-2.07
	5->1 years prior to index date	3.02	2.17-4.21	2.94	2.11-4.11
	≤1 year prior to index date	9.23	5.93-14.37	9.17	5.88-14.28
**DF40-48**	**Overall**	2.74	2.15-3.50	2.69	2.11-3.45
Neurotic, stress-related and	10->5 years prior to index date	1.19	0.81-1.76	1.14	0.77-1.69
somatoform disorders	5->1 years prior to index date	3.48	2.46-4.93	3.40	2.39-4.82
	≤1 year prior to index date	8.14	4.59-14.43	8.21	4.62-14.59
**DF50-59**	**Overall**	1.80	0.65-4.95	1.79	0.65-4.95
Behavioral syndromes associated with	10->5 years prior to index date				
physiological disturbances and physical	5->1 years prior to index date	3.75	1.01-13.97	3.87	1.04-14.48
factors	≤1 year prior to index date	3.00	0.42-21.30	2.93	0.41-20.92
**DF60-69**	**Overall**	1.68	0.87-3.23	1.61	0.84-3.11
Disorders of adult personality and	10->5 years prior to index date	1.13	0.44-2.88	1.08	0.42-2.76
behavior	5->1 years prior to index date	1.00	0.32-3.10	0.93	0.30-2.91
	≤1 year prior to index date	3.75	1.01-13.97	3.70	0.99-13.84
**DF99**	**Overall**	3.39	1.83-6.28	3.36	1.81-6.25
Unspecified mental disorder	10->5 years prior to index date	2.25	0.95-5.34	2.09	0.87-5.00
	5->1 years prior to index date	1.33	0.41-4.33	1.40	0.43-4.57
	≤1 year prior to index date				

Incidence rate ratios (IRRs) for young onset Alzheimer's disease are presented by subcategories of mental and behavioral disorders in 3 time-intervals prior to diagnosis with 95% confidence intervals (CI). The adjusted IRRs are adjusted for age, sex, highest attained educational level at age 40 years (or at time of diagnosis if age at diagnosis <40 years), and civil status at index date. Where no estimates are presented, there were too few events to analyze.

ICD: International classification of diseases

* Excluding mild cognitive impairment and dementia diagnoses
